# Morphological and Immunohistochemical Aspects with Prognostic Implications and Therapeutic Targets of Primary Sinonasal Mucosal Melanoma: A Retrospective Study

**DOI:** 10.3390/cancers16162863

**Published:** 2024-08-16

**Authors:** Cornelia Marina Trandafir, Raluca Maria Closca, Marioara Poenaru, Oana Silvana Sarau, Cristian Andrei Sarau, Marina Rakitovan, Flavia Baderca, Laurentiu Vasile Sima

**Affiliations:** 1 ENT Department, University of Medicine and Pharmacy “Victor Babes”, 300041 Timisoara, Romania; trandafir.cornelia@umft.ro (C.M.T.); poenaru.marioara@umft.ro (M.P.); 2 Department of Pathology, Emergency City Hospital, 300254 Timisoara, Romania; baderca.flavia@umft.ro; 3 Department of Microscopic Morphology, University of Medicine and Pharmacy “Victor Babes”, 300041 Timisoara, Romania; marina.rakitovan@umft.ro; 4 ENT Department, Emergency City Hospital, 300254 Timisoara, Romania; 5 Hematology Department of the Municipal Emergency Clinical Hospital, 300254 Timisoara, Romania; oana.sarau@umft.ro; 6 Faculty of Medicine, University of Medicine and Pharmacy “Victor Babes”, 300041 Timisoara, Romania; csarau@umft.ro; 7 Internal Medicine Department of the Municipal Emergency Clinical Hospital, 300254 Timisoara, Romania; 8 Oro-Maxillo-Facial Surgery Clinic of the Emergency City Hospital, 300062 Timisoara, Romania; 9 Department of Surgery, University of Medicine and Pharmacy “Victor Babes”, Eftimie Murgu Square No. 2, 300041 Timisoara, Romania; sima.laurentiu@umft.ro; 10 Department of Surgery, Emergency City Hospital, Gheorghe Dima Square No 5, 300254 Timisoara, Romania

**Keywords:** sinonasal mucosal melanoma, prognostic factors, tumor immune microenvironment, immunohistochemistry

## Abstract

**Simple Summary:**

Sinonasal melanoma represents an aggressive and rare malignant tumor originating in the melanocytes of the sinonasal mucosa. The prognosis of the tumor is unfavorable and the survival rate is very low; therefore, new treatment strategies are being researched for these patients. The aim of this study was to identify all the patients diagnosed with sinonasal mucosal melanoma during a period of 15 years, and quantify the immune cells in the vicinity of the tumor using the usual hematoxylin–eosin staining method and a series of immunohistochemical reactions. Then, we classified mucosal melanomas according to their immune cells into three subtypes and correlated them with patient survival. The presence of certain tumor immune cells (eosinophils, macrophages, natural killer cells, dendritic cells) had an impact on the prognostic and immunotherapy responses of patients with melanoma and on the progression of the disease.

**Abstract:**

Sinonasal mucosal melanoma originates from melanocytes and it is a rare malignancy in the sinonasal tract. It is an aggressive melanocytic neoplasm with a very poor prognosis. The symptoms are nonspecific and the diagnosis is delayed, usually until the advanced stages of the disease. The current study performs a correlation between the histopathological aspects of sinonasal mucosal melanoma and different types of immune cells present in the microenvironment, with prognostic and therapeutic implications. The endpoint is to quantify the cellular immune microenvironment and correlate it with patient survival. This study presents nine cases of primary sinonasal mucosal melanomas diagnosed at the Emergency City Hospital Timisoara, Romania during a period of 15 years. The histopathological examination was performed in the Department of Pathology of the same hospital, using morphological hematoxylin–eosin staining. Additional immunohistochemical reactions were performed to confirm the diagnosis and evaluate the components of the tumor immune microenvironment. This study identifies eosinophils, macrophages, natural killer cells and plasma cells as favorable prognostic factors. Therefore, a CD8:CD4 ratio of more than 3 is correlated with a good response to PD-1 inhibitor therapy.

## 1. Introduction

Sinonasal melanoma is a rare and highly aggressive tumor originating in melanocytes that derive from neural crest cells. It constitutes 0.5–2% of all melanomas and 4% of melanomas of the head and neck region [1,2,3]. The overall incidence is estimated at 0.5 to 0.71 per million inhabitants per year [4]. Both sinonasal melanoma and its cutaneous counterpart are currently showing an increased number of new cases per year worldwide. A slightly increased incidence is observed in males in the Caucasian population, the fifth and sixth decades being the most affected [5,6,7].

The pathogenesis of sinonasal mucosal melanoma is poorly understood. Sun exposure and ultraviolet light are not risk factors, unlike cutaneous melanoma [8]. Possible etiological factors include chronic smoking, occupational exposure to toxic formaldehyde substances and human papilloma virus infection. None of these factors are present in the etiopathogenesis of cutaneous melanoma, suggesting the involvement of some different pathogenic triggering mechanisms for sinonasal melanoma [9].

The genetic mutations of sinonasal melanoma do not overlap with those observed in cutaneous melanoma, with most studies indicating significant differences in its biological behavior. BRAF and KIT proto-oncogene mutations are rarely involved in the pathogenesis of the mucosal melanoma, being found in only 4% of cases. Thus, sinonasal melanomas are commonly driven by RAS, especially NRAS mutations, and very rarely display BRAF pathogenic variants [10].

Sinonasal mucosal melanomas are usually present with nonspecific symptoms like epistaxis, rhinorrhea, nasal discharge, hyposmia, facial pain and partial or complete nasal obstruction. Advanced stage tumors may present skin ulceration, ophthalmoplegia or exophthalmia, facial paresis or greater weight loss. Initially, the tumor has slow progression, followed by rapid growth in the paranasal sinuses or nasal cavity. Most tumors originate from the septum or lateral walls of the nasal cavity. In advanced stages, a tumor mass protruding through the nasal fossae can be clinically detected [11,12].

Due to the nonspecific symptomatology, patients with sinonasal melanoma are frequently categorized in an advanced stage of the disease [13,14]; 5% of patients have locoregional lymph node metastases at initial presentation and 10–15% of patients have distant metastases at diagnosis, respectively; 40–70% develop distant metastases during the course of the disease [9,15]. The most common sites of distant metastasis are the lungs, liver, bone and brain [9,15,16].

Due to the low incidence of sinonasal melanoma, there is no universal consensus regarding staging, management and treatment protocols, which are often compared with those of cutaneous melanoma. Data published in the English literature related to the prognostic factors of sinonasal mucosal melanoma are based on case reports or small series extended over several decades in order to maximize the number of cases [4].

By analogy with the cutaneous counterpart, prognostic factors include the age of onset, gender of the patients, tumor location, histological type, stage of the tumor and status of resection margins [9,17,18]. However, the prognostic significance of these factors has not been consistently demonstrated across large series.

The neoplastic cells interact with the tumor microenvironment, represented by immune cells, stromal cells, endothelial cells and pericytes. This interaction is crucial for the control of the development and progression of tumors [19]. Tumor-infiltrating lymphocytes (TILs) have been identified in primary tumor tissue, metastatic lymph nodes and distant metastases of mucosal melanoma. TILs represent intra- and peritumoral lymphocytes and are associated with survival benefit [20,21,22,23]. The study of the tumor microenvironment can help us to understand the pathogenic mechanisms of sinonasal mucosal melanoma and can lead to the improvement of targeted therapies.

The mucosal melanoma of the sinonasal tract has a very high mortality rate, with a median survival rate of 30 months and a low 5-year survival rate of 22%. Surgery is considered the mainstay of treatment for primary sinonasal mucosal melanomas, while radiotherapy has a role in postoperative local control of the disease [9].

Current therapeutic strategies aim to improve the prognosis of patients with sinonasal melanoma and represent targeted therapies. As a result, research of the biological mechanisms that trigger and support the carcinogenesis of sinonasal melanoma and identification of its histological substrate that contributes to cell proliferation, invasion and migration are imperatively necessary.

The aim of this study is to present the histopathological aspects of sinonasal mucosal melanoma, to identify and quantify the main compartments of the tumor immune microenvironment and to highlight the main prognostic factors using an extensive panel of immunohistochemical markers.

## 2. Materials and Methods

### 2.1. Patient Selection and Inclusion Criteria

This study was retrospective, with chronological extension over a period of 15 years, and included all cases of sinonasal mucosal melanoma diagnosed between January 2009 and December 2023. The identification of the cases was performed using the specimen reception registers of the Department of Pathology of Timisoara’s Emergency City Hospital. The cases were integrated into the clinical context using the computer database of the hospital. For each patient, age, sex, alcohol or tobacco consumption, symptoms, time span until presentation, site and size of the lesion, endoscopic aspects, surgical procedure, recurrence and oncological treatment were identified. The data related to the survival of the patients were obtained from the Central Register of the Population Records. The inclusion criteria for this study were patient’s age over 18 years, sinonasal mucosa as primary tumor, diagnosis confirmed by histopathological examination and paraffin block available sufficient for immunohistochemical staining.

### 2.2. Ethical Considerations

This study was conducted relying on the ethical guidelines of the Declaration of Helsinki and Romanian legislation. The presence of the annex that accompanied the biopsy and the informed consent of the patient was secured. The histological slides were processed in accordance with the recommendations enforced by the Ministry of Health based on international protocols. The Institutional Review Board Statement No. E-3314/10.06.2024 was approved by the ethical commission of Timisoara’s Emergency City Hospital.

### 2.3. Laboratory Technique

The harvested specimens were fixed in 4% *v*/*v* buffered formaldehyde, and four-micrometer-thick sections were cut using a semi-automated Leica RM2235 rotary microtome (Leica Biosystems, Nussloch, Germany) and displayed on SuperFrost™ microscope slides (St. Louis, MO, USA). In order to obtain the morphological diagnosis, the hematoxylin–eosin technique was primarily used. The histological observations were recorded for each tumor as follows: surface epithelium ulceration, architectural pattern of growth, cell type, melanin pigment, necrosis, inflammatory response, angiolymphatic invasion, perineural invasion, bone or soft tissue invasion by the tumor cells, mitotic figures (number of mitotic figures per 10 high-power fields), atypical mitotic figures, nucleoli, intranuclear cytoplasmic inclusions and assessment of the margins of resection.

The histopathological diagnosis was completed using immunohistochemical reactions. All steps of the immunohistochemical staining were performed with the Leica Bond-Max automatic device (Leica Biosystems Melbourne Pty Ltd., Waverley, Australia), in accordance with the manufacturer’s standardized protocol. All the antibodies and reagents for immunohistochemical reactions were purchased from Leica Biosystems, New Castle, UK. The specific data on the antibodies used in the immunohistochemical study, regarding the type, clone, dilution and exposure time, are presented in Table 1.

Amelanotic tumors required immunohistochemical markers to identify the cellular origin and a panel of melanocytic antibodies were used (anti-S100 protein, anti-HMB45, anti-Melan A and anti-SOX10). In undifferentiated tumors, additional markers were used to exclude other cellular origins (anti-smooth muscle actin, anti-desmin, anti-vimentin, anti-cytokeratin AE1/AE3, anti-synaptophysin and anti-chromogranin). In all cases, an extensive panel of antibodies was used to identify and quantify the components of the tumor immune microenvironment. Thus, for the evaluation of the immune infiltrate, antibodies of lymphoid origin were used (anti-common leukocyte antigen (LCA), anti-CD3, anti-CD4, anti-CD8, anti-CD20, anti-CD138, anti-CD56, anti-CD68 and anti-CD1a).

Three high-power fields were randomly selected from each section in order to evaluate the tumor microenvironment.

According to the location of the immune cell infiltration, the tumors were divided into three immunotypes (type A with loss of immune cells, type B with the immune cells mainly concentrated in the peritumoral stroma and perivascular tissue and type C with diffuse immune cell infiltration). The immunohistochemical profile was completed with antibodies anti-CD117, anti-p53 and anti-Ki67.

### 2.4. Endpoints

The primary endpoint was cellular quantification of the tumor immune microenvironment, and the secondary was to evaluate the association between overall survival and main descriptive findings

### 2.5. Statistical Plan

Data analysis was conducted in R software version 4.3.2 using the following packages: “Survival”, “survminer”, “Rcpp”, “Knitr”, “dplyr”, “ggplot2” and “tibble”. For the ordinal variables, the median was calculated. The percentages were used to present the descriptive data. The Kaplan–Meier curve was generated for survival analysis*,* and the log-rank test was applied for the association between the immunotherapy used and the survival rates. The Chi-squared statistic had statistical significance if the *p*-value was under 0.05.

## 3. Results

### 3.1. Subjects

**Figure 1:**
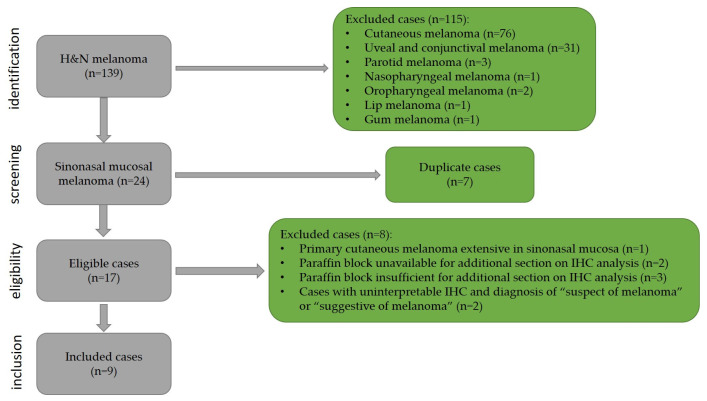
Flowchart of the case selection.

The sample selected for this study had 9 patients with sinonasal melanoma out of 139 patients with head and neck melanomas. Figure 1 shows the diagram with the selection of the cases.

### 3.2. Demographic and Clinical Data

The peak incidence was in the 6th decade and the median age was 67. A female predominance was noticed, with an incidence of 56% (n = 5). A total of 89% of the patients (n = 8) were smokers, while 1 was associated with alcohol consumption. No patient had professional exposure to toxic agents. The most common symptoms were epistaxis, nasal obstruction and hyposmia. The average time span until presentation was five months.

All patients underwent nasal endoscopy and posterior rhinoscopy with identification of a nasal polypoid tumor mass, with a median diameter of 1.2 cm. A presumptive diagnosis of a sinonasal tumor mass was established in all cases. Three patients presented black pigmented lesions, with a major clinical suspicion of melanoma.

The most common site of the above-mentioned lesions was the right nasal fossa (56%, n = 5), followed by the left nasal fossa (22%, n = 2) and, lastly, the nasal septum (11%, 1 case). In one case (11%), the mass had a large extension with localization in the right naso-orbito-sphenoidal mucosa. All demographic data, and some clinical aspects, are presented in Table 2.

The patients underwent computed tomography or magnetic resonance imaging of the head and neck regions in order to evaluate the locoregional tumor extension and clarify the clinical staging. A biopsy of the tumor mass was performed for all patients.

### 3.3. Morphological and Immunohistochemical Aspects

The microscopic examination by hematoxylin–eosin staining revealed a malignant tumor with epithelioid cells and cyto-nuclear atypia in eight cases (89%), two cases (22%) being associated with plasmacytoid features. The predominant pattern was nested (44%, n = 4), followed by the peritheliomatous/perivascular pattern (22%, n = 2) and papillary pattern (22%, n = 2). One case presented a mixed pattern, both nested and fascicular, and mixed cellularity, having an epithelioid and an interspersed fusiform cell population. Only four tumors (44%) showed intracytoplasmic melanic pigment, the rest being achromatic lesions (Table 3).

**Figure 2:**
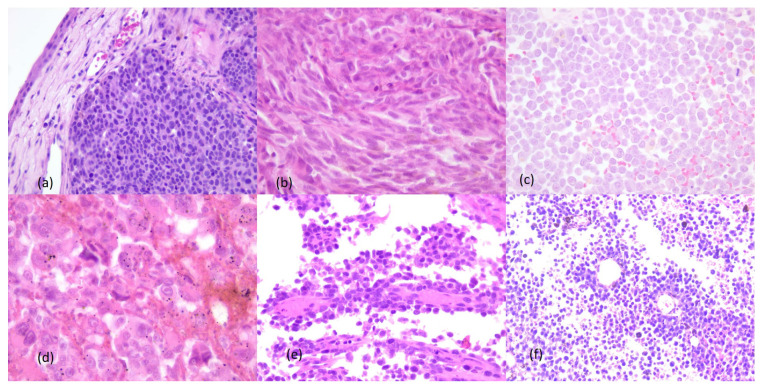
Microscopic aspects after hematoxylin–eosin staining of the sinonasal mucosal melanoma: (**a**) epithelioid cells, nested growth pattern, ob. 10×; (**b**) mixed cellularity, fusiform and nested growth pattern, ob. 40×; (**c**) epithelioid cells with plasmacytoid features, ob. 20×; (**d**) anaplastic cells, ob. 40×; (**e**) papillary growth pattern, ob. 5×; (**f**) peritheliomatous growth pattern, ob. 5×.

Cellular atypia was moderate in most cases. The median value of mitotic index was 7 mitoses on 10 microscopic fields at 400× magnification, while 2 cases (22%) presented poorly differentiated tumors with cyto-nuclear anaplasia, bizarre nuclei and numerous atypical mitotic figures. Figure 2 presents representative microscopic images after hematoxylin–eosin staining of the tumors.

**Figure 3:**
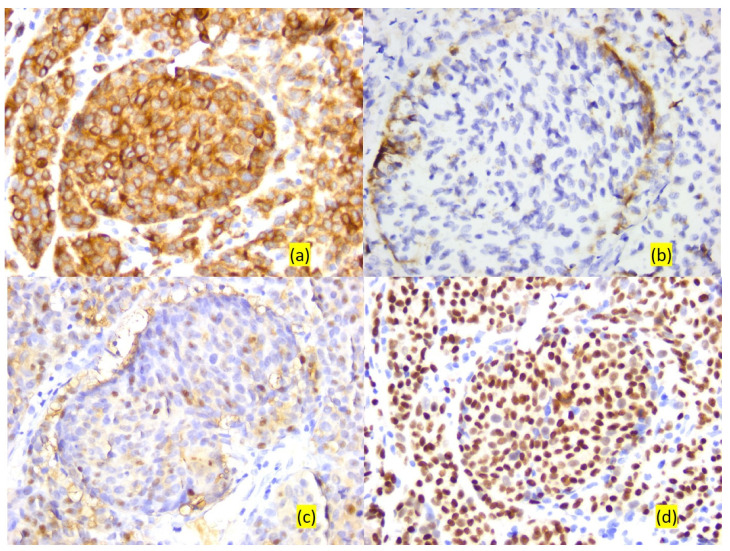
Microscopic aspects. Immunoreactivity of tumor cells to melanocytic markers: (**a**) Melan A, ob. 20×; (**b**) HMB45, ob. 20×; (**c**) S100 protein, ob. 20×; (**d**) SOX 10, ob. 20×.

For the cases of anaplastic tumors and/or achromic ones, an immunohistochemical study was initially required to establish the cellular origin. Thus, epithelial, mesenchymal, muscle and melanocytic markers were used, all five tumors being immunohistochemically positive for melanocytic markers such as HMB45 (45–100% of tumor cells, with moderate and/or intense reaction), S100 protein (50–90% of tumor cells, with moderate and/or intense reaction), Melan A (70–100% of tumor cells, with moderate and/or intense reaction) and SOX 10 (30–100% of tumor cells, with weak, moderate or intense reaction) (Figure 3 and Table 4).

Only four tumors showed extensive areas of ischemic and tumoral necrosis, and ulceration of the lining sinonasal mucosa. Meanwhile, all tumors had rich intratumoral vascularization and numerous intratumoral extravasated erythrocytes. Invasion of blood and lymphatic vessels was observed in six cases, while perineural invasion was present in only one case, which also presented the invasion of the underlying cartilage.

Evaluation of the resection margins was possible in six cases (67%), the other three cases being tumor fragments from incisional biopsy. All six cases of excisional biopsy presented free surgical margins, with oncological clearance. For these cases, pTNM was classified according to the American Joint Committee on Cancer staging system. Thus, five tumors (56%) were staged as pT3, and the tumor with the invasion of the underlying cartilage was staged as pT4a. One case also had a regional lymph node biopsy (jugular/carotid lymph node), with the present metastasis being staged as pN1, the rest of the tumors having a stage of pNx. Table 5 highlights the main prognostic histopathological aspects after hematoxylin–eosin staining.

The immunohistochemical study identified and quantified the tumor microenvironment, highlighting the inflammatory component. The immune tumor microenvironment revealed the presence of a cellular inflammatory infiltrate, and classified tumors according to Immunoscore into three categories. Therefore, five cases (56%) had immunotype B, three cases (33%) had immunotype A and only one case (11%) was classified with immunotype C.

**Figure 4:**
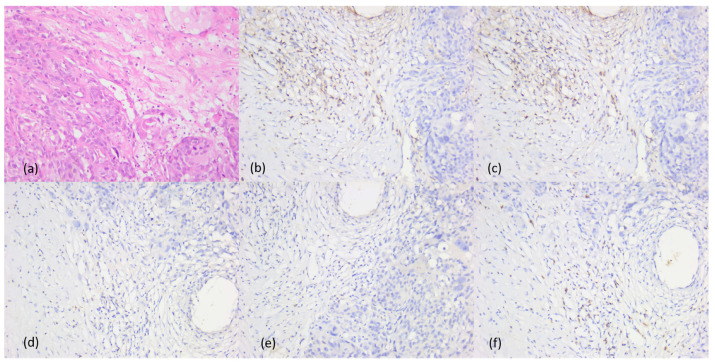
Tumor immunotype A. Morphological aspects and immunohistochemical profile, ob. 20×: (**a**) morphological aspects, hematoxylin–eosin staining; (**b**) LCA positive reaction in immune cells; (**c**) CD20+ B lymphocytes; (**d**) CD3+ T lymphocytes; (**e**) CD4+ cytotoxic T lymphocytes; (**f**) CD8+ helper T lymphocytes.

Immunotype A tumors showed rare peri- and intratumoral inflammatory cells, predominantly CD20+ B lymphocytes, a few CD4+ and CD8+ T lymphocytes, with CD4:CD8 ratio = 1:4 and neutrophil/lymphocyte ratio = 0 (Figure 4).

**Figure 5:**
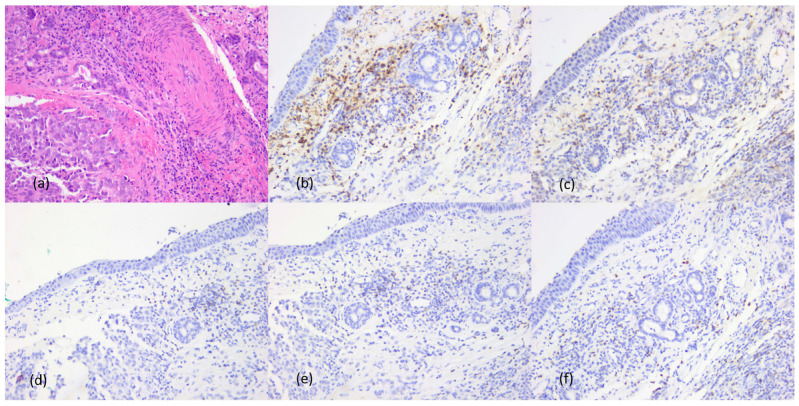
Tumor immunotype B. Morphological aspects and immunohistochemical profile, ob. 20×: (**a**) morphological aspects, hematoxylin–eosin staining; (**b**) LCA positive reaction in immune cells; (**c**) CD20+ B lymphocytes; (**d**) CD3+ T lymphocytes; (**e**) CD4+ cytotoxic T lymphocytes; (**f**) CD8+ helper T lymphocytes.

Immunotype B tumors had predominantly perivascular and/or peritumoral stromal inflammatory infiltrate and predominantly CD20+ B lymphocytes, but also numerous CD8+ T lymphocytes, with a CD4:CD8 = 1:4 or 1:3 ratio and a neutrophil granulocyte/leukocyte ratio of <1. Increased amounts of plasma cells and macrophages were also observed (Figure 5).

**Figure 6:**
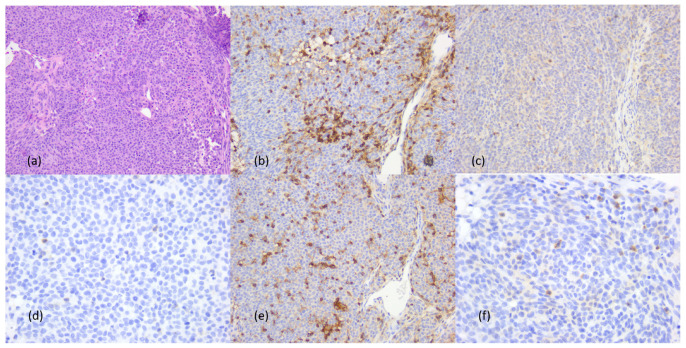
Tumor immunotype C. Morphological aspects and immunohistochemical profile, ob. 20×: (**a**) morphological aspects, hematoxylin–eosin staining; (**b**) LCA positive reaction in immune cells; (**c**) CD20+ B lymphocytes; (**d**) CD3+ T lymphocytes; (**e**) CD4+ cytotoxic T lymphocytes; (**f**) CD8+ helper T lymphocytes.

Immunotype C showed diffuse inflammatory cell infiltration with predominance of CD4+ T lymphocytes, CD4:CD8 ratio = 2:1 and neutrophil/lymphocyte ratio = 2, with peritumoral predominance of neutrophils and eosinophils. Rare plasma cells and macrophages were observed in peri- and intratumoral inflammatory infiltrates (Figure 6). The entire immunohistochemical profile of the inflammatory component can be found in Table 6.

**Figure 7:**
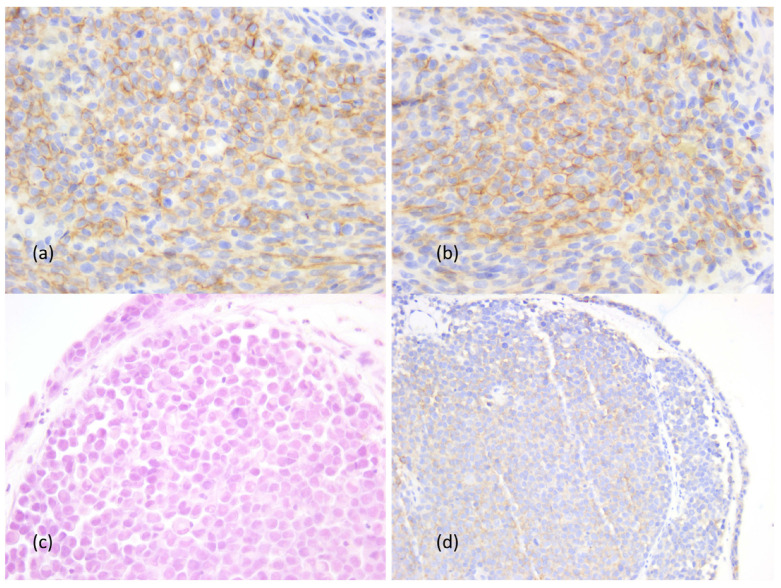
Aberrant reaction of CD56 and CD138 in the tumor cells. (**a**,**b**) Tumor cells positive for CD56 and CD 138, ob. 20×; (**c**,**d**) tumor with plasmacytoid features, ob. 20× and aberrant CD56 positive reaction, ob. 10×.

CD56+ NK lymphocytes had a very low rate, with the median value of 0.1%, the higher rate being present in immunotype B tumors. It was found that two cases had a weak positive CD56 reaction in 40% of the tumor cells, one of them also associating with a CD138 positive reaction in the tumor cells. In addition, another case presented an aberrant positive CD138 reaction in plasmacytoid tumor cells. The inflammatory infiltrate did not show CD138+ immune cells in any sinonasal mucosal melanoma (Figure 7).

Immunotype B tumors had peritumoral and perivascular cell infiltrates rich in CD68+ macrophages (the median value was 5%), and in plasma cells the median value was 10%. In immunotype C, an absence of CD68+ macrophages and a depletion of plasma cells were noted.

All cases of sinonasal melanoma showed few CD1a+ dendritic cells, with a median of 0.1%. There were increased reactions in immunotype B, while, in immunotype A, they were rare or absent.

Six cases (67%) were positive at CD117, with a median value of 0.1%, with weak and moderate reaction, and three cases (33%) were negative. Also, p53 protein presented a heterogeneous reaction in most cases (67%, n = 6), with weak or moderate/intense positive immunoexpression in 40–85% of tumor cells. In three cases (33%), no p53 overexpression was noted.

The Ki67 proliferation index had a median value of 20%. The Ki67 index was increased (≥40%) in four cases, most of them being type A and one type B. Two sinonasal melanoma had an intermediate Ki67 index (20–40%) and three tumors had a low Ki67 index (<20%).

### 3.4. Survival Analysis

**Figure 8:**
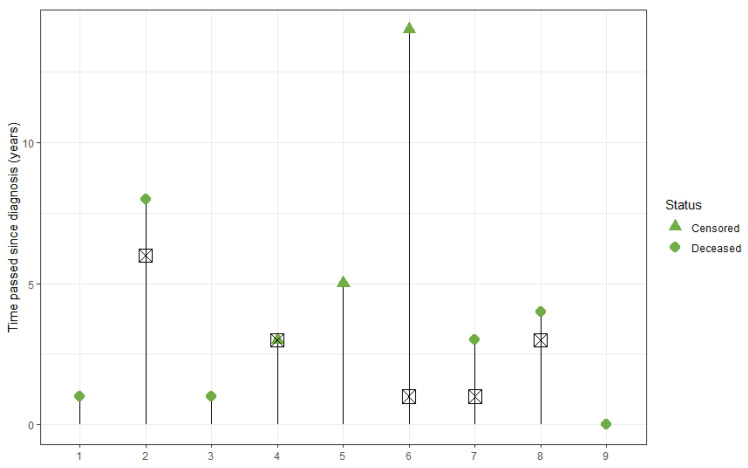
Graphical representation of each patient’s survival time, with their respective status. The square crosses represent the moment of the disease’s relapse.

At the last follow-up, three patients were still alive 3 years, 5 years and, respectively, 14 years since the diagnosis of the disease (data that can be observed in Figure 8, together with the time of disease relapse).

**Figure 9:**
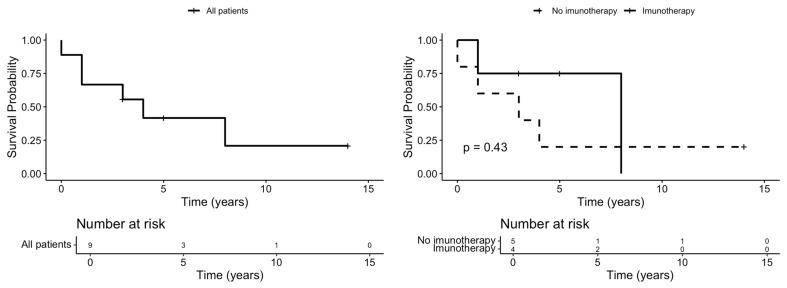
Survival curves of the entire patient group (**left**); survival curves and comparison between the patients with immunotherapy (continuous line) and those without (dashed line) (**right**).

The median overall survival (OS) time of the entire group was 4 years, with a mean survival of 3 years. The OS at 1, 3, 5 and 10 years was 67%, 56%, 42% and 21%, respectively (Figure 9, left).

Four patients (cases 1, 2, 4 and 5) benefited from immunotherapy. In the group that received immunotherapy, two events occurred (50% of the patients deceased), while in the group that did not benefit from it there were four observed events (80%). The log-rank test was utilized to assess the differences between the survival rates of the two groups. The Chi-squared statistic was 0.6, with a *p*-value of 0.43 (Figure 9, right).

Table 7 presents the overall survival of the patients grouped by pigmentation, eosinophiles, macrophages, plasma cells, dendritic cells, NK lymphocytes, immunotype and immunotherapy.

## 4. Discussion

Sinonasal melanomas are rare tumors with a small number of cases presented in the English literature. The present study highlighted the experience of a fifth important hospital by number of cases in Romania during the last 15 years. Nine cases of sinonasal melanomas were diagnosed. All the cases showed epithelioid cells, five out of nine being achromic tumors.

Melanoma is one of most aggressive malignant neoplasms, arising from the uncontrolled proliferation of melanocytes, with a five year survival rate of only 25% when diagnosed at an advanced metastatic stage [24]. This aggressive behavior and high capacity to disseminate develops drug resistance and prevents immunosurveillance, all of which depend on the heterogeneity of the tumor tissue and the tumor microenvironment. The tumor microenvironment includes extracellular matrix molecules, neoformation vessels and stromal cells, including immune cells, endothelial cells, pericytes, fibroblasts, activated adipocytes and mesenchymal stem cells. The cellular components of the tumor microenvironment are characterized by great phenotypic plasticity supported by their own interconnections and malignant cells, and are involved in progression regulation, resistance to targeted therapy and immunosurveillance of the melanoma [25,26,27].

Currently, the tumorigenesis of mucosal melanoma is known as a process that originates from multiple genetic and epigenetic alterations in the neoplastic cells. However, numerous steps in carcinogenesis, such as cell proliferation, invasion, angiogenesis and metastasis, are modulated by components of the tumor microenvironment. Between these components are temporal and spatial interconnections that lead to a biosystem with a major impact on the tumor process [23,28,29].

The tumor immune microenvironment is an important component of the large tumor microenvironment and plays a major role in supporting tumor growth and progression. The immune-stimulating cells promote the anti-cancer immune response and include tumor-infiltrating lymphocytes (TILs), natural killer (NK) lymphocytes, neutrophils, eosinophils and dendritic cells. The immunosuppressive component inhibits the anti-cancer immune response in order to facilitate the progression of the tumor, and consist of regulatory T cells, mast cells, cells and macrophages [20,23,28].

TILs are defined as lymphocytes within and around cancer cells and have been associated with survival benefit. The significance of TILs in cutaneous melanoma has been studied, but there is insufficient research on TILs in sinonasal melanoma. In the literature, we identified only four retrospective studies with small groups that highlighted the role of TILs in the carcinogenesis of sinonasal melanoma. Moreover, in a retrospective cohort study, Yin et al. analyzed the correlation between TILs and prognosis, concluding that TILs can be used to predict the prognosis for patients with sinonasal melanoma [21]. They showed that TILs were independent factors for progression-free survival, but there was no clear correlation with overall survival. Also, CD8+ T cells and NK cells were highly expressed in patients with no disease progression and cases with diffuse distribution and high density of inflammatory cells had a better prognosis [21].

Ledderose et al. classified patients according to the density of TILs into two categories, brisk and non-brisk, and showed that brisk TILs were associated with lower T3 stage and increased recurrence-free and 5-year survival, and patients survived significantly longer than those without lymphocytic infiltrates. These results indicate that TIL density is a strong prognostic factor for better survival in patients with sinonasal melanoma and suggest that prospective studies with larger cohorts are warranted to determine whether TILs should be included in the future in the American Joint Committee on Cancer staging guidelines [30]. In another study, it was established that melanomas with a high density of CD3 and CD4 positive lymphocytes had a positive prognosis for survival [31]. In addition, TILs may be predictive markers for efficacy of immunotherapy [20,32], and TIL therapy has been demonstrated to improve the survival and outcome of melanoma [32,33]. These authors classify TILs in three groups: A (small number of lymphocytes), B (moderate to severe TILs disposed in perivascular and peritumoral patterns) and C (severe TILs disposed and diffused peri- and intratumorally) and correlate these groups with overall survival, the patients with C-type TILs having the best overall survival. In the present study, the overall survival was better in patients with B-type TILs (80%, 60%, 60% and 30% at 1, 3, 5 and 10 years). The patient with C-type TILs deceased 4 years after the diagnosis, this characteristic being correlated with the high percentage of neutrophils and a high neutrophil/lymphocyte ratio.

CD8+/CD4+ TIL ratio is associated with the response to PD-1 inhibitor treatment in solid cancers. Regarding mucosal melanoma patients, ratios of CD8+/CD4+ lower than 2 predicted a lack of response to treatment, while ratios higher than 2.7 had an 81.3% response rate. In addition, the presence of more than 1900/mm^2^ of CD8+ T cells in the melanoma predicted a better response to therapy [34]. Between the studied patients, three out of nine received PD-1 inhibitor treatment. For these patients, the CD8+/CD4+ ratio was 4, and the overall survival was 4.5 and 14 years, two of them being free of disease.

Currently, there are no specific studies for the role of B cells in sinonasal mucosal melanoma. The data presented were extrapolated from data found on the cutaneous counterpart. In melanoma, up to 33% of immune cells can be CD20+ B cells, which sustain inflammation processes and may represent a predictor factor for survival and response to immunotherapy [35,36,37]. Actual data on the role of tumor-associated B cells are divergent. Most studies show that tumoral and peritumoral infiltrating B cells are positively correlated with favorable survival, while other studies find no significant association between B cell infiltration and overall patient survival, or even a correlation between B cell infiltration, with a worse prognosis. These significant differences may arise from the design of the experimental studies or may reflect the presence of immunosuppressive regulatory B cells [37,38,39,40,41].

In the carcinogenesis of the melanoma, the B lymphocytes could play a pro- and an anti-tumoral role. Also, CD20+ B cells can conform tumor-associated tertiary lymphoid structures that modulate T cell activation. Antibodies expressed by B cells mediate tumor progression and some isotypes have a strong anti-tumor immune response. Therefore, B lymphocytes represent a potential immunotherapy target in the context of checkpoint inhibitors [36,37]. In the last decade, immunotherapy has at least revolutionized the management of cutaneous melanoma. Recent studies show that CD20+ B cells may not be passive bystanders of checkpoint inhibitor immunotherapy and B cell phenotypes are correlated with a positive response. CD20+ B cells contribute to the formation of tumor tertiary lymphoid structures, facilitating the induction of T cells required for the response to checkpoint inhibitor immunotherapy. In contrast, specific B cell subsets often correlate with immune-related adverse events in cancer immunotherapy. Thus, this multifaceted role of B-cell immunity will lead to new therapeutic strategies and biomarkers that can be translated into the clinic to improve the therapeutic effects of checkpoint inhibitors in melanoma [35,36,37,38,39,40,41].

Natural killer cells are a component of the innate immune system; they detect and target malignant cells, playing multiple roles in anti-tumor immune surveillance. They produce pro-inflammatory cytokines, are able to produce lysis of malignant cells through the secretion of perforin and granzyme B, and create apoptosis by the activation of caspase [42]. NK lymphocytes have a crucial role in the recruitment of the dendritic cells and in the maturation of the tumor microenvironment [43,44]. Because of these roles, the NK cells represent an attractive target for immunotherapy in melanomas by blocking inhibitory receptors expressed by these innate immune cells. However, melanomas can develop mechanisms to avoid NK cell anti-tumoral responses, such as secreting anti-inflammatory cytokines, upregulating ligands for inhibitory receptors, and recruiting immunosuppressive cells [42]. Between the studied patients, two cases showed the presence of NK cells in the tumor microenvironment. Between these, NK cells were accompanied by the dendritic cells in one patient, who responded very well to immunotherapy, with a five year survival rate, and who is still alive at the moment of writing this article.

Some studies show the presence of intratumoral CD56+ NK cells in the melanoma microenvironment [45,46], but other data suggest that intratumoral NK cell infiltration is very low [22,47]. Although the number of NK cells is not associated with survival, the density and distribution of immune cell infiltration influence the progression of the disease in patients with sinonasal melanoma. Therefore, patients with a high expression of natural killer T cells have a positive benefit in controlling the progression of the disease [21]. In the present study, the identification of NK cells more than 0.5% in the tumor microenvironment, observed in two cases, was corelated with an 8 and, respectively, 5 year survival, greater than in other cases included in this study. Similar data have also been observed by other authors [21,42].

The other important immune components are dendritic cells, which derive from myeloid precursors. They promote and coordinate the immune system against tumoral antigens, having the capacity to coordinate the innate and the adaptive immune systems. The dendritic cells represent CD1a positive antigen presenting cells, which interact with T cells throughout the major histocompatibility complex. As part of the innate system, dendritic cells produce cytokines and chemokines and play an important anti- or pro-inflammatory role, according to the stimuli received from the microenvironment [48]. Data suggest that there is a decrease in number and function of dendritic cells in melanoma, with a deeper decrease in metastatic tumors than in localized tumors [49,50]. Even if the number of studied cases was small, in eight out of nine cases, there were no dendritic cells observed. The only case that presented dendritic cells was the patient who responded very well to immunotherapy, with a 5 year survival interval. These data are similar to those published by other authors [48,49,50].

Melanoma is considered an immunogenic tumor, with the most impressive results from immunotherapy; hence, dendritic cells are the key step to these effects. Type 1 dendritic cells have been of interest in the last decade due to their specialized ability to directly activate CD8+ T cells [51]; numerous therapeutic strategies have targeted these immune cells in the treatment of melanoma [52]. The absence of dendritic cells in the tumor microenvironment can lead to the resistance of immunotherapy. Mucosal melanoma is also able to avoid the complex mechanism of T cell activation by influencing maturation. Therefore, tumor cells can produce inhibitory cytokines and create an unfavorable environment for the maturation of dendritic cells, leading to a decreased immune response [53,54].

Macrophages and neutrophils are also therapeutic targets in melanoma treatment. Tumor-associated macrophages belong to stromal cells and are abundant in the tumor microenvironment, being associated with poor clinical outcomes [55]. There is evidence that macrophages are involved in tumor progression through multiple mechanisms. They create an immunosuppressive microenvironment by producing cytokines, chemokines and growth factors, which modulate the recruitment of immune cells and inhibit anti-tumor responses. They also play a role in angiogenesis through the production of pro-angiogenic factors and matrix metalloproteinases and vascular construction that provide the source of oxygen and nutrients to malignant cells. Macrophages play an important role in dissemination and metastasis, contributing to the invasion, extravasation, survival, intravasation and colonization of tumor cells [56]. In contrast to these data, the presence of macrophages was observed in seven out of nine cases, with overall survival of 71%, 57%, 57% and 29% at 1 year, 3 years, 5 years and 10 years, respectively.

Tumor-associated neutrophils originate from myeloid precursors and represent the most abundant population of inflammatory cells produced in the first responders of innate immunity. They contribute to tumor progression and metastatic dissemination, showing pro-tumor function by stimulating the extracellular matrix and acute inflammation. Neutrophils contain multiple intracytoplasmic granules with proteases, such as matrix metalloprotease 9 and neutrophil elastase, which they release into the tumor microenvironment, and can remodel the extracellular matrix and promote tumor invasion. They also produce immunosuppressive factors, including arginase 1 and transforming growth factor beta, which are involved in suppressing adaptive immunity and tumor progression [57,58]. In the present article, the presence of neutrophils was correlated with 80%, 60%, 60% and 30% at 1 year, 3 years, 5 years and 10 years, respectively. These data are in contrast to the data found in the English literature.

Superposed on the neutrophils’ presence are the eosinophils, which are also correlated with a better prognosis and long overall survival.

In skin melanoma, an important factor in the tumor microenvironment is the plasma cell. Few data about the presence of plasma cells in the mucosal melanomas have been published [29,33]. In the present article, the plasma cells were correlated with better prognosis, with 67%, 50%, 50% and 25% at 1 year, 3 years, 5 years and 10 years, respectively.

Recent studies show that the neutrophil/lymphocyte ratio is a prognostic factor for the progression of the localized melanoma and for metastatic dissemination [59,60]. Lino-Silva et al. performed a large retrospective study and reported that overall survival at 5 years was 63% for melanomas with a ratio under 2 and 53% for the neutrophil/lymphocyte ratio over 2 group [59]. Ma et al. analyzed the impact of the neutrophil/lymphocyte ratio in patients with locoregional metastases using the cut-off of 2.5 and showed that the disease-free survival rate of the low value group was significantly higher compared with that of the high value group. They concluded that the neutrophil/lymphocyte ratio over 2.5 was a strong predictor for disease recurrence in patients with stage III melanoma [60]. In the studied group, similar to the medical literature, a neutrophil/lymphocyte ratio equal to 2 was observed in only one case of a young woman, 35 years old at the time of diagnosis, who deceased four years from the day of presentation after presenting two relapses of the disease at the second and third year after the established diagnosis.

Currently, the gold standard in the diagnosis of sinonasal melanoma remains the microscopic examination after hematoxylin–eosin staining correlated with immunohistochemical reactions. As in other pathologies, even in the cutaneous counterpart, different biomarkers are used in establishing the prognosis and treatment of patients with sinonasal melanoma. Thus, morphological and immunohistochemical studies could be completed with molecular analyses to identify genetic abnormalities such as NRAS and c-KIT mutations [61,62]. Also, the circulating tumor DNA levels can be directly correlated with tumor progression and can be detectable for selective genes by quantitative and digital polymerase chain reactions (PCR), such as digital droplet PCR, along with next generation sequencing techniques [63,64,65].

## 5. Conclusions

This study highlighted favorable prognostic factors in sinonasal melanomas, the presence of melanin in the cytoplasm of the tumor cells and immunotype B of TILs. The presence of NK cells, eosinophils, macrophages and plasma cells increased the overall survival, while dendritic cells were correlated with unfavorable prognosis. A CD8:CD4 ratio more than 3 showed a good response to PD-1 inhibitor treatment. The data identified in this study highlight new horizons for finding targeted therapies in the treatment of patients with sinonasal mucosal melanoma.

## Figures and Tables

**Table 1:** Data related to the antibodies used for immunohistochemical reactions.

Antibody	Substrate	Clone	Dilution
HMB45 ^1^	Monoclonal, mouse	HMB45	1:60
Melan A	Monoclonal, mouse	A103	1:50
S100 protein	Polyclonal, rabbit	EP32	1:100
SOX10 ^2^	Monoclonal, rabbit	SP267	1:100
CK AE1/AE3 ^3^	Monoclonal, mouse	AE1/AE3	1:100
Vimentin	Monoclonal, mouse	V9	1:800
Desmin	Monoclonal, mouse	DE-R-11	1:75
Smooth muscle actin	Monoclonal, mouse	Asn-1	1:50
Synaptophysin	Monoclonal, mouse	27G12	1:200
Chromogranin	Monoclonal, mouse	5H7	1:200
CD117 ^4^	Monoclonal, rabbit	EP10	1:200
LCA ^5^	Monoclonal, mouse	X16/99	1:40
CD3 ^6^	Monoclonal, mouse	LN10	1:500
CD4 ^7^	Monoclonal, mouse	4B12	1:100
CD8 ^8^	Monoclonal, mouse	4B11	1:500
CD20 ^9^	Monoclonal, mouse	L26	RTU ^10^
CD56 ^11^	Monoclonal, rabbit	MRQ-42	RTU
CD138 ^12^	Monoclonal, mouse	Mi15	RTU
CD68 ^13^	Monoclonal, mouse	514H12	1:100
CD1a ^14^	Monoclonal, mouse	MTB1	RTU
p53 protein	Monoclonal, mouse	DO-7	1:800
Ki67 index ^15^	Monoclonal, mouse	MM1	1:200

^1^ HMB45 (human melanoma black); ^2^ SOX10 (Sry-related HMg-Box gene 10); ^3^ CK AE1/AE3 (pan cytokeratin AE1/AE3); ^4^ CD117 (cluster of differentiation 117); ^5^ LCA (leucocyte common antigen); ^6^ CD3 (cluster of differentiation 3); ^7^ CD4 (cluster of differentiation 4); ^8^ CD8 (cluster of differentiation 8); ^9^ CD20 (cluster of differentiation 20); ^10^ RTU (ready-to-use); ^11^ CD56 (cluster of differentiation 56); ^12^ CD138 (cluster of differentiation 138); ^13^ CD68 (cluster of differentiation 68); ^14^ CD1a (cluster of differentiation 1a); ^15^ Ki67 (index of cell proliferation Ki67).

**Table 2:** Demographic data and clinical aspects of sinonasal melanoma.

Case	Age	Sex	Signs and Symptoms	Onset of Symptoms	Site	Size	Observations
1	67	M	Epistaxis	4 months	Left nasal fossa	0.9 cm	Smoker
2	74	M	Nasal obstruction, hyposmia	9 months	Right nasal fossa	1.4 cm	Smoker
3	69	M	Epistaxis	3 months	Right nasal fossa	0.5 cm	Smoker
4	73	F	Epistaxis, nasal obstruction	5 months	Right nasal fossa	2 cm	Smoker
5	59	F	Epistaxis, nasal obstruction	1 month	Left nasal fossa	1.2 cm	Non-smoker
6	67	F	Epistaxis, hyposmia, nasal obstruction	3 months	Right nasal fossa	0.9 cm	Smoker
7	77	F	Nasal obstruction, nasal discharge	4 months	Nasal septum	1.6 cm	Smoker
8	35	F	Epistaxis, oral respiration, snoring	2 weeks	Right nasal fossa	1.1 cm	Smoker
9	59	M	Nasal obstruction, rhinorrhea, hyposmia	12 months	Right naso-orbito-sphenoidal area	2.3 cm	Smoker and Alcoholic

**Table 3:** The cellularity, growth pattern and melanin distribution after hematoxylin–eosin staining.

Case	Cells	Pattern	Melanin
1	Epithelioid	Nested	+++ ^1^
2	Epithelioid	Nested	Absent
3	Epithelioid	Papillary	Absent
4	Epithelioid	Nested	+++
5	Epithelioid and fusiform	Mixed: nested and fascicular	Absent
6	Epithelioid and plasmacytoid	Papillary	+ ^2^
7	Epithelioid	Peritheliomatous	Absent
8	Epithelioid	Nested	Absent
9	Epithelioid and plasmacytoid	Peritheliomatous	+++

^1^ +++ (intense reaction); ^2^ + (weak reaction).

**Table 4:** Immunohistochemical reactions of melanocytic markers.

Case	HMB45		S100 Protein		Melan A		SOX 10	
1	Ratio	Intensity	Ratio	Intensity	Ratio	Intensity	Ratio	Intensity
2	90%	++ ^1^	90%	+++ ^2^	70%	++/+++ ^3^	100%	+++
3	100%	+++	85%	++/+++	95%	+++	100%	+++
5	50%	++	80%	++/+++	90%	++/+++	90%	++
7	90%	+++	50%	+++	70%	+++	30%	+/++ ^4^
8	45%	++/+++	80%	+++	100%	+++	100%	+++

^1^ ++ (moderate reaction); ^2^ +++ (intense reaction); ^3^ ++/+++ (moderate/intense reaction); ^4^ +/++ (weak/moderate reaction).

**Table 5:** Histopathological aspects after H&E staining with prognostic role.

Case	Ulceration	Necrosis	Mitotic Index ^1^	VL ^2^	Pn ^3^	Resection Margins	pTNM ^4^
1	Absent	Absent	6	V0L1	Pn0	Negative	pT3Nx
2	Absent	Absent	5	V0L0	Pn0	NA ^5^	NA
3	Absent	Absent	15	V0L1	Pn0	NA	NA
4	Present	Present	9	V0L0	Pn0	Negative	pT3Nx
5	Present	Present	5	V0L1	Pn0	Negative	pT3Nx
6	Absent	Absent	7	V0L1	Pn0	Negative	pT3N1
7	Absent	Absent	7	V0L1	Pn0	Negative	pT3Nx
8	Present	Present	16	V0L0	Pn0	NA	NA
9	Present	Present	9	V0L1	Pn1	Negative	pT4aNx

^1^ Number of mitoses on 10 high-power fields (HPF); ^2^ vascular and lymphatic invasion; ^3^ perineural invasion; ^4^ TNM pathological staging; ^5^ not applicable.

**Table 6:** Immunohistochemical profile of the tumoral immune infiltrate.

Case	LCA *	CD20+ B Cells **	CD3+ T Cells **	CD4+ T Cells **	CD8+ T Cells **	CD56+ NK Cells **	CD68+ Macrophages *	CD1a+APC *	CD117+ Mast Cells *	Other Inflammatory Cells	Immunotype
1	75%	65%	10%	5%	20%	<0.1%	0.5%	<0.1%	<0.1%	10% plasma cells, 10% neutrophils and eosinophils *	B
2	70%	75%	4.5%	5%	15%	0.5%	10%	<0.1%	<0.1%	15% plasma cells, 5% neutrophils and eosinophils *	B
223	90%	85%	<0.1%	3%	12%	0%	5%	<0.1%	0%	5% plasma cells	A
4	95%	70%	5%	5%	20%	0%	5%	<0.1%	0%	-	A
5	70%	70%	4.5%	5%	20%	0.5%	10%	0.5%	<0.5%	15% plasma cells, 4% neutrophils and eosinophils *	B
6	70%	70%	5%	5%	20%	0.1%	5%	<0.1%	0.5%	20% plasma cells, 4.5% neutrophils and eosinophils *	B
7	70%	70%	5%	5%	20%	0.1%	10%	0.5%	0.5%	15% plasma cells, 4% neutrophils and eosinophils *	B
8	90%	55%	<0.1%	30%	15%	<0.1%	0.5%	<0.1%	<0.1%	30% neutrophils, 0.5% plasma cells	C
9	90%	70%	5%	5%	20%	0%	10%	0%	0%	-	A

* Positive reaction of inflammatory infiltrate; ** positive reaction of lymphocytes.

**Table 7:** Overall survival of the patients.

Variable	1 Year OS	3 Years OS	5 Years OS	10 Years OS
Pigmentation −	60%	40%	20%	-
Pigmentation +	75%	75%	75%	37.5%
Eosinophiles −	50%	50%	-	-
Eosinophiles +	80%	60%	60%	30%
Macrophages −	50%	50%	-	-
Macrophages +	71%	57%	57%	29%
Plasmacytes −	67%	67%	-	-
Plasmacytes +	67%	50%	50%	25%
Dendritic cells −	57%	57%	38%	19%
Dendritic cells +	100%	50%	50%	-
NK lymphocytes −	57%	43%	21%	21%
NK lymphocytes +	100%	100%	100%	-
Immunotype A	33.3%	33.3%	-	-
Immunotype B	80%	60%	60%	30%
Immunotype C	100%	100%	-	-
Immunotherapy −	60%	40%	20%	20%
Immunotherapy +	75%	75%	75%	-

− absent; + present.

## Data Availability

The data that support the fundings on this study are available from the corresponding author upon reasonable request.
